# Supplementary material for: Predicting in-hospital mortality in ICU patients with Coronary heart disease and diabetes mellitus using machine learning models
Source: PLoS One. 2025 Aug 14;20(8):e0330381. doi: 10.1371/journal.pone.0330381 (PMC12352758; doi:10.1371/journal.pone.0330381)
Supplement: S2 — (PDF) [file pone.0330381.s002.pdf]

CITI PROGRAM

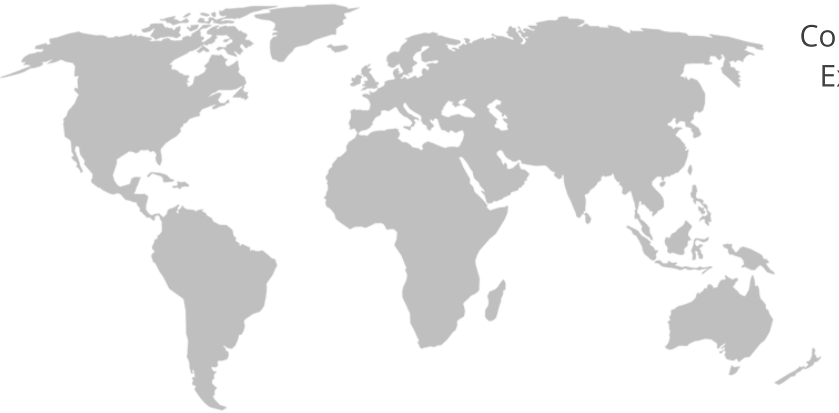

Completion Date 16-Oct-2024
Expiration Date 16-Oct-2027
Record ID 65828445

This is to certify that:

guang tu

Not valid for renewal of certification through CME.

Has completed the following CITI Program course:

Human Research
(Curriculum Group)
Data or Specimens Only Research
(Course Learner Group)
2 - Refresher Course
(Stage)

Under requirements set by:

Massachusetts Institute of Technology Affiliates

CITI
Collaborative Institutional Training Initiative

101 NE 3rd Avenue, Suite 320
Fort Lauderdale, FL 33301 US
www.citiprogram.org

Generated on 04-Jan-2025. Verify at www.citiprogram.org/verify/?w104a7cfa-e9f9-4a62-bfaa-44d6464a681b-65828445
