# Supplementary material for: Predicting in-hospital mortality in ICU patients with Coronary heart disease and diabetes mellitus using machine learning models
Source: PLoS One. 2025 Aug 14;20(8):e0330381. doi: 10.1371/journal.pone.0330381 (PMC12352758; doi:10.1371/journal.pone.0330381)
Supplement: Attachment — Submitted filename: Response_to_Reviewers_auresp_2.docx [file pone.0330381.s005.docx]

**Journal Requirements:**

1. If the reviewer comments include a recommendation to cite specific previously published works, please review and evaluate these publications to determine whether they are relevant and should be cited. There is no requirement to cite these works unless the editor has indicated otherwise.

**Additional Editor Comments:**

I am really grateful to review this manuscript. In my opinion, this manuscript can be published once some revision is done successfully. I made two suggestions and I would like to ask your kind understanding.

**Firstly, it can be noted that experts use impurity/permutation importance for testing the strength of association between the dependent variable and its major predictor then they employ the SHAP summary/dependence plot for evaluating the direction of the association. In this context, I would like to ask the authors to derive impurity/permutation importance as well. Secondly, it can be noted that boosting and the random forest often register similar performance outcomes (84%-85%) but bring different results in permutation importance and SHAP plots. Boosting focuses on the final decision of the strongest tree with minimal bias whereas the random forest focuses on the majority vote of all trees with minimal variance. Both models have their own strengths and weaknesses, hence which model performs and explains better depends on various conditions. In this vein, I would like to ask the authors to (1) compare their strengths and weaknesses and (2) compare their permutation importance and SHAP plots in a comprehensive manner.**

**Response:**

Thank you very much for your valuable comments and suggestions. We have carefully considered your feedback and have made the necessary revisions to our manuscript. Below are our responses to the specific points you raised:

**1. Response to the First Point**

We have now included the calculation of permutation importance for both the Gradient Boosting Classifier and the Random Forest Classifier. As requested, we have also provided a detailed comparison of the permutation importance and SHAP plots for these two models.

1.1 Permutation Importance: This method measures the decrease in model performance (AUC) when the values of a single variable are randomly shuffled. We have calculated the permutation importance for the top 10 variables for both the Gradient Boosting Classifier and the Random Forest Classifier. The results are presented in Figures 5a and 5c, respectively. These figures show the relative importance of each variable in predicting in-hospital mortality.

1.2 SHAP Summary Plots: To evaluate the directionality of the associations, we have generated SHAP summary plots for both models. Figures 5b and 5d display the SHAP summary plots for the Gradient Boosting Classifier and the Random Forest Classifier, respectively. These plots not only validate the key variables identified by the Boruta algorithm but also provide insights into the direction of the associations between the variables and the outcome.

**2. Response to the Second Point**

We have conducted a comprehensive comparison of the strengths and weaknesses of the Gradient Boosting Classifier and the Random Forest Classifier, as well as their permutation importance and SHAP plots.

2.1 Comparison of Model Strengths and Weaknesses:

(1) Gradient Boosting Classifier:

Strengths: Gradient Boosting focuses on the final decision of the strongest tree with minimal bias. This makes it highly effective in capturing complex interactions and non-linear relationships in the data. It achieved the highest AUC value of 0.8532 in our study, demonstrating its superior ability to distinguish between high-risk and low-risk patients.

Weaknesses: Gradient Boosting can be more prone to overfitting if not properly tuned. It also requires careful parameter tuning and can be computationally intensive.

(2) Random Forest Classifier:

Strengths: Random Forest relies on the majority vote of all trees with minimal variance, making it robust against overfitting. It achieved an AUC of 0.8461, which is slightly lower than Gradient Boosting but still very competitive. Random Forest is also less sensitive to hyperparameters and faster to train.

Weaknesses: While Random Forest is less prone to overfitting, it may not capture complex interactions as effectively as Gradient Boosting. It can also be less interpretable due to the large number of trees involved.

2.2 Comparison of Permutation Importance and SHAP Plots:

(1) Permutation Importance:

Gradient Boosting Classifier: The permutation importance plot (Figure 5a) shows that variables such as age, BUN, and pH have the highest importance scores. This indicates that these variables have a strong association with in-hospital mortality.

Random Forest Classifier: The permutation importance plot (Figure 5c) also highlights age, BUN, and pH as important variables, but the relative importance scores differ slightly from those of the Gradient Boosting Classifier. This suggests that while both models identify similar key variables, their relative importance may vary.

(2) SHAP Summary Plots:

Gradient Boosting Classifier: The SHAP summary plot (Figure 5b) shows the directionality of the associations. For example, higher values of age and BUN are associated with increased mortality risk, while higher pH values are associated with lower risk.

Random Forest Classifier: The SHAP summary plot (Figure 5d) provides similar insights but with slight differences in the magnitude and direction of the effects. This highlights the different ways in which the two models interpret the same variables.

In conclusion, both Gradient Boosting and Random Forest classifiers have their own strengths and weaknesses, and their performance and interpretability depend on the specific characteristics of the dataset. We believe that our comprehensive analysis provides valuable insights into the predictive power and interpretability of these models, which can guide clinicians in selecting the most appropriate model for their needs.

Thank you once again for your constructive feedback. We have made significant improvements to our manuscript based on your suggestions, and we believe that these revisions have strengthened our study.

**Reviewer #1: We do not deny the authenticity of the data provided by the MIMIC.‌However, the phenomenon mentioned in the May 2025 Science article （O'Grady C. Low-quality papers surge thanks to public data and AI. Science. 2025 May 22;388(6749):807-808. doi: 10.1126/science.adz1715. Epub 2025 May 22. PMID: 40403045.）is “authors limited their analysis to certain years, or certain ages of people in the survey. That suggests the authors were on the hunt for statistically significant results to generate easy publications, Spick says. But fishing for results in such a huge data set is bound to come up with many false positive findings. When the team took a closer look at the 28 NHANES studies that had explored depression, they found that only 13 of the results survived a statistical adjustment that corrects for the risk of finding false positives.”**

**So we hope the author can provide the initial data of 4366 cases extracted from the MIMCI database, so that other researchers can verify whether there is selection bias or false positive issues in repeating this study without specific data screening.**

**Perhaps with initial data, I can provide a good explanation and validation for the 1-3 questions I raised during my first review.**

**1.Unclear inclusion and exclusion criteria can impact sample size and outcomes. Therefore, it is crucial to clarify key factors such as MIMIC database version, age, and diabetes classification to ensure accurate sample size and consistent research baseline.**

**2.The exclusion criteria only mention CHD patients admitted to the ICU. How should data from CHD patients with multiple ICU admissions be handled? Additionally, how does a change in sample size affect the selection of key variables in the machine learning model?**

**3.The MIMIC database includes clinical data such as vital signs, laboratory results, clinical scores, complications, medication usage, interventions, and outcomes. The exclusion criteria mentioned in the text only state "lack of key clinical data (e.g., vital signs or laboratory results at admission)" without providing original**

**data to verify potential bias in the exclusion criteria. This lack of transparency may affect the sample size included**

**Response:**

Thank you very much for your continued interest and valuable feedback on our manuscript. We appreciate your concerns regarding the potential for selection bias and false positives in our study, especially in light of the issues highlighted in the May 2025 Science article.

In response to your request, we are pleased to confirm that we are willing to provide the initial data of the 4366 cases extracted from the MIMIC-IV database. This will allow other researchers to verify whether there are any selection biases or false-positive issues when repeating this study without specific data screening. We believe that sharing our initial dataset will enhance the transparency and reproducibility of our research, and we are committed to facilitating further validation and scrutiny of our findings.

We understand the importance of addressing these concerns comprehensively. By providing the initial data, we hope to offer a clear and detailed explanation for the questions you raised during your first review. We are confident that this will help to further validate our study and address any potential issues related to data selection and analysis.

Thank you once again for your thorough review and constructive suggestions. We are committed to ensuring that our research meets the highest standards of scientific integrity and reproducibility.
